# Prevalence and Factors Associated With Suicidal Ideation Among Children and Adolescents Attending a Pediatric HIV Clinic in Uganda

**DOI:** 10.3389/fsoc.2021.656739

**Published:** 2021-06-15

**Authors:** Justine Diana Namuli, Joyce Sserunjogi Nalugya, Paul Bangirana, Etheldreda Nakimuli-Mpungu

**Affiliations:** ^1^ Department of Psychiatry, College of Health Sciences, Makerere University, Kampala, Uganda; ^2^ Mulago National Referral and Teaching Hospital, Ministry of Health, Kampala, Uganda

**Keywords:** suicidal ideation, pediatric HIV, Uganda, children, adolescents

## Abstract

**Background:** Suicidal behavior and HIV/AIDS are vital public health challenges especially in low and middle-income countries. As suicide in adults is perturbing for those closest to them, this sentiment is much more intense and generalized in the case of a child or adolescent. Knowledge of factors associated with suicidal ideation in HIV infected children and adolescents may inform suicide prevention strategies needed to improve their quality of life. This study aimed to assess the prevalence and factors associated with suicidal ideation among HIV infected children and adolescents attending a pediatric HIV clinic in Uganda.

**Methods:** Data from a sample of 271 children and adolescents aged 6–18 years living with HIV/AIDS attending a pediatric HIV clinic was analyzed. Child characteristics and clinical variables were assessed using a socio-demographic questionnaire and medical records respectively. Suicidal ideation and depression were assessed using the Child Depression Inventory. The types of behavioral problems and the parent–child relationship were assessed using Child Behavioral Check List (6–18 years) and the Parent Child Relationship Scale respectively. Child exposure to different stressful life events was assessed with a series of standardized questions. Logistic regression models were used to explore factors independently associated with suicidal ideation.

**Results:** The prevalence of suicidal ideation was 17%. In the multivariate analysis; Child exposure to family or friend’s death (prevalence rate ratio (PRR = 2.02; 95% CI, 1.01–4.03), *p* = 0.046), HIV wasting syndrome (PRR = 0.39; 95% CI, 0.21–0.75, *p* = 0.04), Depression (PRR = 1.08; 95% CI, 1.03–1.12, *p* = 0.001), Anxiety symptoms (PRR = 1.10; 95% CI, 1.01–1.20, *p* = 0.024) and Rule breaking behavior (PRR = 1.06; 95% CI, 0.99–1.13, *p* = 0.051) were independently associated with suicidal ideations.

**Conclusion:** The prevalence of suicidal ideation among children and adolescents living with HIV/AIDS is substantial. Children and adolescents with exposure to family or friend’s death, those with higher depression scores, anxiety symptoms and rule breaking behavior are more likely to report suicidal ideation. Those with HIV wasting syndrome were less likely to report suicidal ideation. There is urgent need for HIV care providers to screen for suicide and link to mental health services.

## Introduction

Globally suicide is the second leading cause of death for young people ages 10–34 years and accounts for 20% of all deaths annually^1^. Similarly, 1.8 million children ages 0–15 years worldwide are infected with HIV/AIDS of which Sub-Saharan African countries including Uganda carry the largest burden of these pediatric HIV infections (UNAIDS, 2020). Furthermore, suicidal behavior which includes both suicidal ideation and suicidal attempt is one of the psychiatric problems associated with HIV/AIDS(3). Thus, suicidal behavior and HIV infection coupled together are public health challenges.

Different studies showed that the global prevalence of suicidal ideation and attempts among HIV positive youth varied across the world. For instance, the prevalence estimate of suicidal ideation has been reported to range from 10 to 26% (Cheung and Dewa, 2006; Martinez et al., 2009; Arseniou et al., 2014; Bolakale et al., 2016; Smith Fawzi et al., 2016; Woollett et al., 2017), while the prevalence estimate of suicide attempt has been reported to range from 1.3 to 20% (Cheung and Dewa, 2006; Hidaka et al., 2008; Mutumba et al., 2015; Woollett et al., 2017). In Uganda, the few studies on suicidal behavior in persons living with HIV/AIDS have reported the following prevalence rates: 17.1% for the 12-month prevalence of attempted suicide rate among HIV positive adolescent ages 10–18 years (Musisi and Kinyanda, 2009), 7.8% for moderate to high risk suicidality and 3.9% of life-time attempted suicide among HIV adult patients(13). Further, rates of suicidal ideation of 8.8 and 3.1% for suicide attempts among patients 15 years and above were reported by Rukundo et al. (2016). All these Ugandan studies were conducted among majorly adult population, and a few in adolescents, however children below 10 years were not included in the studies. The factors that have been reported to be associated with suicide ideation and attempts among adults and youth living with HIV/AIDS have been gender, negative life events, depression (Kinyanda et al., 2012; Arseniou et al., 2014; Wonde et al., 2018), the clinical stage of HIV/AIDS (Wonde et al., 2018), perception of poor physical health, physical pain (Rukundo et al., 2016), poor social support and HIV related stigma (Martinez et al., 2012; Mutumba et al., 2015; Bitew et al., 2016; Wang et al., 2018; Wonde et al., 2018).

According to the Uganda population-based HIV impact assessment (UPHIA) 2016–2017 published in July 2019, approximately 96,000 of Ugandan children (ages 0–14 years) were living with HIV (Ministry of Health, 2019). This corresponded to a prevalence of 0.5%, thus living with such a chronic disease may increase the risk for mental health problems like suicide (Mars et al., 2014). Although suicide is common among HIV positive youth worldwide, there is little report about suicide among HIV infected children and adolescents in Uganda. Knowledge of factors associated with suicidal ideation in these vulnerable children may inform suicide prevention strategies needed to improve their quality of life. Therefore, the aim of this study was to determine the prevalence and factors associated with suicidal ideation among HIV infected children and adolescents aged 6–18 years attending a pediatric HIV clinic in Uganda.

## Methods

### Study Setting and Population

The study was conducted at a pediatric HIV care clinic at Mildmay Uganda, an HIV treatment center in Kampala. Mildmay Uganda is a non-government organization which has been providing specialized, holistic and comprehensive, outpatient care for persons living with HIV (PLWH) since 1998. It provides specialized pediatric palliative care and intensive nutritional rehabilitation for positive children and as well runs a weekly mental health clinic.

### Study Procedure

Study data were collected between January and March 2014. The eligibility criteria required participants to be HIV positive children or adolescents aged 6–18 years, living with the same primary caregiver for at least 12 months before study entry. Individuals who were very sick were excluded from the study. Trained research assistants worked with primary HIV care providers at the treatment center to obtain a register of clients who had come to the clinic on a given day. As clients would be seated in the waiting area waiting for their turn to see the HIV care provider, five names of clients were called out at ago and asked to identify themselves by show of a hand. Research assistants approached these clients, explained study procedures, determined eligibility and administered the study questionnaires to parents/caregivers who provided informed consent and whose children had provided assent.

### Study Measures

#### Outcome Variable

Suicidal ideation was our outcome variable. Suicidal ideation was assessed using item 9 on the Child Depression Inventory (CDI) by Kovacs (1992). The CDI is comprised of 27 items rated on a three-point scale [0 (none) to 2 (distinct symptom)]. In our study, the CDI was administered by the psychiatrist with special training in child and adolescent mental health. Participants that gave an affirmative answer on item 9; which stated: “*I think about killing myself but I would not do it.” And “I want to kill myself.”* thus scoring 1 or 2 on the CDI were regarded to have suicidal ideations.

### Predictor Variables

A socio-demographic questionnaire was used to obtain descriptive information including age, gender, education level, religion and orphan status. Education level attained was categorized into “No formal education”, ‘‘primary education” and or “secondary education”. Religion was categorized into “Christians” and “Non-Christians”. Orphan status was categorized into “Orphan” and “Not orphan”. The second part of the questionnaire asked about child experiences with a significant negative life events like (exposure to family/friend’s death, child sexual abuse, child physical abuse parent separation/divorce, HIV status disclosure, birth of a sibling, change of residence, school related problems, etc.). Participants were required to provide a [YES or NO] answer on occurrence of each negative life event.

### Child Mental Health Variables

***Depression*:** Depression was assessed using the Child Depression Inventory (CDI) scale.

Participants that scored clinical cut-off of >13 were regarded as having significant depression symptoms (Bose et al., 1994).

***Emotional and Behavioral problems*:** Caregivers reported on their child's emotional and behavioral problems using the Child Behavior Checklist for ages 6–18 years. This tool has been validated for use among Luganda speaking children and adolescents with severe malaria by Bangirana et al., 2009 in Uganda (Bangirana et al., 2009). One hundred thirteen items rate the severity of behavioral symptoms categorized into eight syndrome scales (Anxious/Depressed, Withdrawn/Depressed, Somatic Complaints, Social Problems, Thought Problems, Attention Problems, Rule-Breaking Behavior and Aggressive Behavior). Parents rate on a three-point Likert scale from 0 (not true) to 3 (very true) how “true” the items were in describing their child's behavior. The Cronbach’s alphas for the scales ranged from 0.64 to 0.83. The eight syndrome scales have been validated in 30 societies and have proven useful in multicultural assessment of children (Ivanova et al., 2007). The CBCL gives cut-offs for boys and girls in the age ranges 6–11 and 12–18 showing which scores are normal, borderline or of clinical significance. This study utilized cut-offs similar to those used for children in Ethiopia and Algeria (Achenbach and Rescorla, 2001). The cut-offs for scores in the clinical range were at the 97% percentile (Achenbach and Rescorla, 2001) suggesting that 3% of these children had behavioral problems. Scores equal or higher than the lowest score in clinical range were categorized as being of clinical significance. Permission to use the scale was sought from the developers of the scale.

***Parent–child relationship*:** This was assessed with the Parent Child Relationship Scale (PCRS), by Pianta, RC (1992) (Pianta, 1992) which is a self-report instrument for caregivers acting in a parental role. Two subscales were used: Conflict (UNAIDS, 2020), Positive aspect of relationship. Each item is rated on a five-point scale (1 = definitely does not apply to 5 = definitely applies). The PCRS generates a total scale score reflecting overall relationship. The developer calculated alpha reliability based on 714 subjects, ages 4.5–5.5. Conflicts (alpha = 0.83) and Positive aspects of relationship (alpha = 0.72) (Pianta, 1992). Higher total scores indicated more caregiver involvement, good communication, and youth autonomy, while lower scores indicated poor child-parent relationship which would be a predictor for suicidal ideation. All these tools were translated in the local language Luganda) and have been used in the Ugandan population.

### Clinical Variables

The study participant’s medical records were reviewed to record participants’ most recent CD4 counts (at 6 months) and WHO clinical stage of their HIV disease and any comorbidities or opportunistic infections.

### Ethical Considerations

The study was approved by the School of Medicine Research and Ethics Committee at Makerere University, Mildmay Uganda Institutional Review Committee and the Uganda National Council for Science and Technology. Assent was obtained from each participant and written informed consent from the primary care giver. Confidentiality of the information collected was assured to the participants at all stages of the data collection process.

### Statistical Analysis

Data was analyzed using STATA version 12. The goal of the analyses was to estimate and identify, the prevalence of suicidal ideation among the HIV infected children and adolescents and which child psychological, social and clinical factors were associated with suicidal ideation.

Initially, a binary variable was created for suicidal ideations, with the variable coded 1, for presence of suicidal Ideations and coded 0, for absence of suicidal ideations. Due to violations of normality in the dependent variable, we opted to use general linear model with the Poisson regression to evaluate bivariate and multivariate associations between suicidal ideations and other study variables. Variables significant at *p*-value ≤ 0.20 in the unadjusted analysis were included in the final multiple logistic regression analysis in which variables with a *p*-value ≤ 0.05 at a 95% confidence interval were considered as statistically significant. Both forward and backward selection of variables was carried out using this final model.

## Results

### Characteristics of Study Participants

The study involved a total of 271 HIV-infected children and adolescents. The participant’s ages ranged from 6 to 18 years, with a mean age of 11.6 years; SD 3.48. There was a female preponderance (151, 55.7%) among the participants. The majority of study participants (98.5%) had attained some level of education, but at the time of the interview, (5.5%) were not in school, the commonest reason being ill-health (5.9%). One hundred seventy-five (64.6%) had been orphaned, one hundred nine (40.22%) had HIV clinical stage III and IV disease, prior to starting antiretroviral therapy (ART). The Most recent CD4 count among the participants ranged from 8 to 3,336 cells/mm^3^ with mean of 811.9 cells/mm^3^ SD 499.7 and majority of the patients 238; 87.8% were on ART. The detailed socio-demographic characteristics of the study sample are shown in Table 1.

**TABLE 1:** Socio-demographics characteristics of the study participants (*N* = 271).

Characteristics	Total (*N* = 271) n(%)
**Age category**	
<13	160(59.04)
≥13	111(40.96)
**Sex**	
Female	151(55.72)
Male	120(44.28)
**Orphan status**	
Not orphan	96(35.42)
Orphan	175(64.58)
**Religion**	
Christians	218(80.44)
Non-Christians	53(19.56)
**Education status**	
No formal education	4(1.48)
Primary	219(80.81)
Secondary	48(17.71)
**Child HIV status disclosed**	
Yes	161(59.71)
No	110(40.59)

The prevalence of suicidal ideation among the 271 participants was **17%.**

### Factors Associated With Suicidal Ideation Among Human Immunodeficiency Virus Infected Children and Adolescents at Bivariate and Multivariate Analysis

#### Socio-Demographic Characteristics

Among the socio demographic factors, children who had attained a primary level of education were more likely to report suicidal ideations than those in secondary, (*p* = 0.013). Education level lost its significance at multivariate analysis. Gender and orphan status did not show any associations.

#### Significant Negative Life Events

The children who had knowledge of their HIV status (*p* = 0.013), those who had exposure to family or friend’s death (*P* < 0.0001) those who had experienced sexual trauma *p* < 0.0001) and school related problems (*p* = 0.057) were more likely to report suicidal ideations, though at multivariate analysis it’s only **child exposure to family or friend’s death** that remained independently associated with suicidal ideation; [adjusted Prevalence rate ratio (**aPRR) = 2.02, 95%CI = 1.01 to 4.03.,** *p* **-value = 0.046].**

#### Clinical Variables

During bivariate analyses, the children and adolescents with late WHO HIV disease stage (III andIV) were more likely to report suicidal ideations (*p* = 0.122) than those in early stage (I andII). Likewise, those with CD4 count lower than the sample mean CD4 count of 811 cells/mm^3^ were more likely to report suicidal ideations, however all these associations lost their significance during multivariate analyses.

Children with **HIV wasting syndrome** were less likely to report suicidal ideations and this maintained its significance at multivariate analysis [adjusted Prevalence rate ratio **(aPRR) = 0.39, 95%CI =0.21 to 0.75,** *p* **-value = 0.004].**

#### Parent-Child Relationship

Children and adolescents who obtained higher total scores on the parent child relationship scale (PCRS), thus indicating more caregiver involvement, good communication, and youth autonomy, were more likely not to report suicidal ideations (mean 71.64; SD = 10.15) when compared with those with lower scores (Mean 66.83; SD = 12.64) with a mean difference of 4.81, 95%CI = 1.4 to 8.2, *p*-value = 0.005, though parent child relationship lost its significance at multivariate analysis.

#### Depression

Children and adolescents who obtained higher depressive scores were more likely to report suicidal ideations, (Mean 19.11; SD = 9.77) than those with lower scores, (Mean 8.91; SD = 5.70) with a mean difference of −10.2, 95%CI = −12.3 to −8.1, *p*-value <0.0001. **Depression** maintained its significance at multivariate analysis [adjusted Prevalence rate ratio **(aPRR) =1.08, 95%CI = 1.03 to 1.12,** *p* **-value = 0.001].**

#### Emotional and Behavioral Problems Among the Human Immunodeficiency Virus Infected Children and Adolescents

Children and adolescents who obtained higher scores in anxiety symptoms (*p* < 0.0001), Withdrawal symptoms (*p* < 0.0001), somatic symptoms (*p* < 0.0001), thought problems (*p* < 0.0001), social problems (*p* = 0.208) and rule breaking behavior (*p* = 0.152) were more likely to report suicidal ideations, than those with lower scores. However, at multivariate analysis it’s only **anxiety symptoms**; [adjusted Prevalence rate ratio **(aPRR) =1.10, 95%CI =1.01 to 1.20,** *p* **-value = 0.024]** and **rule breaking behavior (aPRR) =1.06, 95%CI = 0.99 to 1.13,** *p* **-value = 0.051]** that maintained their significance.

Tables 2, 3 summarize the demographic and clinical profiles of study participants associated with suicidal ideations at bivariate analyses. Table 4 summarizes the factors independently associated with suicide ideation among children and adolescents living with HIV.

**TABLE 2:** The demographic and clinical profiles of study participants associated with suicidal ideations at Bivariate analysis (*N* = 271).

Characteristics	Presence of suicidal ideations (*N* = 46) *n* (%)	Absence of suicidal ideations (*N* = 225) *n* (%)	χ2	*p*-value
**Child demographic characteristics**				
Age category (years)				
<13	22 (47.83)	138 (61.33)	2.881	0.090
≥13	24 (52.17)	87 (38.67.2)		
Sex				
Female	28 (60.87)	123 (54.67)	0.596	0.440
Male	18 (39.13)	102 (45.33)		
Religion				
Christians	37 (80.43)	181 (80.44)	0.007	0.935
Non-Christians	9 (19.57)	44 (19.56)		
Education				
Primary	32 (69.57)	191 (84.89)	6.153	0.013
Secondary	14 (30.43)	34 (15.11)		
Child status				
Not orphan	17 (36.96)	79 (35.11)	0.057	0.812
Orphan	29 (63.04)	146 (64.89)		
**Child clinical characteristics**				
HIV disease stage				
Early (I and II)	20 (43.48)	142 (63.11)	6.123	0.013
Late (III and IV)	26 (56.52)	83 (36.89)		
CD4 counts mean (SD)	708.1 (468.7)	833.2 (504.1)	125.1*	0.122
**Negative life events**				
Child knowledge of his/her HIV status				
Yes	35 (76.09)	126 (56.25)	6.238	0.013
No	11 (23.91)	98 (43.75)		
Exposure to family/friend’s death				
Yes	30 (65.22)	65 (28.89)	22.139	<0.0001
No	16 (34.78)	160 (71.11)		
Child sexual abuse or rape				
Yes	13 (28.26)	12 (5.33)	23.975	<0.0001
No	33 (71.74)	213 (94.67)		
Child physical abuse				
Yes	9 (19.57)	52 (23.11)	0.275	0.600
No	37 (80.43)	173 (76.89)		
Change of residence				
Yes	6 (13.04)	27 (12.00)	0.039	0.844
No	40 (86.96)	198 (88.00)		
School related problems				
Yes	21 (45.65)	70 (31.11)	3.621	0.057
No	25 (54.35)	155 (68.89)		
Parental separation/divorce				
Yes	3 (3.69)	7 (3.11)	1.250	0.264
No	43 (93.48)	218 (96.89)		
Birth of a sibling				
Yes	0 (0.00)	4 (1.78)	1.498	0.221
No	46 (100.00)	221 (98.22)		
Exposure to violence				
Yes	6 (13.04)	21 (9.33)	0.586	0.444
No	40 (86.96)	204 (90.67)		
Any other				
Yes	13 (28.26)	29 (12.89)	0.830	0.362
No	33971.74)	196 (87.11)		
**Opportunistic infections**				
HIV wasting syndrome				
Yes	9 (19.57)	72 (32.0)	2.818	0.093
No	37 (80.43).	153 (68.00)		

*The result is a t-test.

**TABLE 3:** Factors associated with suicidal ideations (Depression, Emotional andBehavioral problems and Parent Child relationship) at Bivariate analysis.

Characteristic	Presence of suicidal ideations mean(SD)	No suicidal ideation mean(SD)	Mean difference(95%CI)	*p*-value
Depression	19.11(9.77)	8.91(5.70)	−10.20(−12.3_−8.1)	<0.0001
Parent child relationship	66.83(12.64)	71.64(10.15)	4.81(1.4–8.2)	0.005
Anxiety symptoms	5.83(3.64)	3.25(3.03)	−2.57(−3.6_−1.6)	<0.0001
Withdrawal symptoms	6.83(4.09)	3.89(3.69)	−2.93(−4.1 _−1.7)	0.000
Somatic symptoms	5.74(4.01)	3.67(3.25–4.54)	−2.07(−3.1_−1.0)	<0.0001
Thought problems	4.85(3.27)	2.60(2.94)	−2.24(−3.2_−1.3)	<0.0001
Social problems	5.02(3.43)	4.35(3.26)	−0.67(−1.7–0.4)	0.208
Rule breaking behavior	3.72(4.30)	2.90(3.33)	−8.15(−1.9–0.3	0.152
Aggressive behavior	9.59(6.87)	9.01(5.89)	−0.58(−2.5–1.4)	0.56
Other problems from CBCL	3.67(3.11)	4.41(3.34)	0.74(−0.3–1.8)	0.17
Total CBCL	50.33(22.72)	42.06(49.11)	−8.27(−22.8–6.3)	0.27

**TABLE 4:** Multivariate logistic model. Independent Factors associated with Suicidal ideations among HIV infected Adolescent Children (*N* = 271).

Characteristics	Adjusted prevalence rate ratio(aPRR)	95%CI	*p*-value
Child age category	0.51	0.22–1.19	0.119
Child education	1.74	0.87–3.48	0.114
HIV disclosure status	1.33	0.60–2.98	0.484
Exposure to Family/Friend’s death	2.02	1.01–4.03	0.046
Sexual trauma	0.96	0.52–1.78	0.905
School related trauma	0.85	0.46–1.57	0.601
WHO HIV stage	0.90	0.441.85	0.774
CD4 count	0.99	0.99–1.00	0.772
HIV wasting syndrome	0.39	0.21–0.75	0.004
Depression	1.08	1.03–1.12	0.001
Parental child relationship	1.00	0.97–1.02	0.751
Anxiety symptoms	1.10	1.01–1.20	0.024
Withdrawal symptoms	0.99	0.90–1.10	0.901
Rule breaking behavior	1.06	0.99–1.13	0.051
Somatic symptoms	0.99	0.89–1.10	0.813
Social problems	1.16	0.60–2.25	0.657
Thought problems	1.02	0.92–1.14	0.677

## Discussion

Our main findings were; 1) The prevalence of suicidal ideation among children and adolescents living with HIV/AIDS was 17%; 2) HIV infected children and adolescents with exposure to family or friend’s death, those with higher depression scores, anxiety symptoms and rule breaking behavior scores are more likely to report suicidal ideation; 3) Those with HIV wasting syndrome were less likely to report suicidal ideation.

Our study shows that the prevalence of suicidal ideation among children and adolescents living with HIV/AIDS is substantial, which is more or less similar to rates that have been reported in previous studies among youth and adults with HIV/AIDS such as 16% in Nigeria (8), 15.5% in Thailand (Benjamin Lee and Manik Chhabra, 2011) and 14.0% in Canada (5).

The rate of suicidal ideation obtained in this study is higher and almost twice the rate of suicidal ideation of 8.8% reported by Rukundo and colleagues 2016 in Mbarara, Western Uganda in similar settings (Rukundo et al., 2016). This difference might be due to variations in the study populations. For example, in this current study we assessed children from 6 to 18 years while Rukundo and colleagues involved participants aged 15 years and above. The other reason could be the degree of openness with which children reported their experiences which might not be similar with that of adults. Children because of their immaturity may not know the implications of suicidal behavior just like adults do. In Uganda suicide is a cultural abomination and even legally once someone attempts suicide the law takes charge, so the adults are likely to under report suicidal ideation, yet children may freely express themselves about it.

However, when the rates of suicidal ideation reported in this study are compared to those reported elsewhere, they are much lower. For instance, in Ethiopia Wonde and colleagues 2019 reported prevalence rate of 27.1% among youth with HIV/AIDS(15), similarly higher rates have been reported in other parts of the world: 31.6% in China(17), 27% in the United States (Carrico, 2010), 21% in Australia (Kelly et al., 1998) and 19% in New York (Millett et al., 2012). However, all these studies were conducted either among youth or adults, not children. This makes our study unique for it is the first to our knowledge to assess suicidal ideation among this vulnerable age group of 6–18 years living with HIV/AIDS.

Several previous studies have reported depression to be associated with suicidal behavior and suicide (Kinyanda et al., 2012; Arseniou et al., 2014; Wang et al., 2018). Similarly, our study found both anxiety and depression to be independently associated with suicidal ideations. Seemingly, anxiety, not just depression, was independently associated with suicidal ideation. Those children and adolescents living with HIV/AIDS who got higher scores in depression and anxiety symptoms were more likely to report suicidal ideations. However, research on anxiety being independently associated with suicidal ideation is still inconclusive, for instance O’Neil Rodriguez and Kendall 2014 results support an independent relationship between anxiety symptomatology and suicidal ideations among youth 7–17 years, after controlling for depressive symptoms (O'Neil Rodriguez and Kendall, 2014). Furthermore, there are some studies that have supported an independent association between anxiety symptomatology and suicidal ideation beyond demographic factors and depression (Gould et al., 1998; Boden et al., 2007) whereas findings of other studies have not supported an independent relationship (Esposito and Clum, 2002; Foley et al., 2006). Thus, the relationship between anxiety disorders and suicidality among children and adolescents warrant further exploration in studies using exhaustive and continuous measures of suicidality.

Interestingly, our study also shows that HIV infected children and adolescents with aggressive behavior were more likely to report suicidal ideation. Few studies have explored the relationship between aggression and suicidal behavior in children and adolescents. A meta-analysis of 4,693 children and adolescents conducted by Hartley and colleagues 2018 reported a statistically significant relationship between reactive aggression and suicidal behaviors (Hartley et al., 2018). Our finding is also consistent with a study by Hill and colleagues 2020 who also found that aggression was significantly associated with child or adolescent suicidal ideation (Hill et al., 2020). However, these studies were done in HIV negative children, which makes our study the first to report that this special group of children and adolescents with HIV who have aggressive behavior are also at increased risk of having suicidal ideation. Previous studies have reported an association between aggressive traits and increased risk for suicidal behaviors in adults (Turecki, 2005; Swogger et al., 2014; Singh and Rao, 2018).

In addition, participants with history of exposure to family/friend’s death were twice more likely to develop suicide ideation than those who had no history of family/friend’s death. Kinyanda and colleagues 2005 (Kinyanda et al., 2005) have reported negative life events in childhood, negative life events later in life, and negative life events in the previous year to be key contributors to suicidal ideation and attempt. Our results are further supported by other studies of HIV populations in Ethiopia (Wonde et al., 2018), South Africa (Petersen et al., 2010) and United States (Small et al., 2014).

Finally, in this current study children and adolescents with HIV wasting syndrome(weight loss ≥10% within 12 months or from baseline visit or weight loss ≥7.5 and 5% within 6–3 months, respectively), were less likely to report suicidal ideations. This finding could probably be due to the fact that by the time a person reaches this stage of HIV wasting syndrome they have gone through the HIV five stages of grief (denial, anger, bargaining, depression and acceptance) and have reached acceptance. This acceptance stage of grief is when one starts to realize that has two options to give up or fight and defeat the disease. Humans being resilient, tend to use different coping mechanisms and dig deep into their reserves of sheer willpower. This alone goes a long way in the fight against the HIV disease which may explain why this group of participants were less likely to report suicidal ideations.

On the other hand, our study among children and adolescents living with HIV/AIDS in 6–18 years age group, did not find gender to be associated with suicidal ideations like it has been found in other studies (Kinyanda et al., 2012; Wonde et al., 2018).

### Limitation of the Study

This being a cross-sectional design, we cannot report the causal relationships of the associations we found. In addition, we cannot rule out social desirability and recall bias as other study limitations.

### Clinical Implications

This study emphasizes the substantial burden of suicidal ideation among children and adolescents living with HIV/AIDS in Uganda which is a major risk factor for completing suicide, and a potentially fatal event. It also identifies and characterizes children with HIV/AIDS who are at greater risk for suicidal ideation.

This implies that clinicians should holistically assess these children who come for HIV care, so that appropriate interventions including early referral for mental health care are made.

## Conclusion

The prevalence of suicidal ideation among children and adolescents living with HIV/AIDS is substantial. Children and adolescents with exposure to family or friend’s death, those with higher depression scores, anxiety symptoms and rule breaking behavior scores are more likely to report suicidal ideation. Those with HIV wasting syndrome were less likely to report suicidal ideation. We recommend further research to explore suicidality and its relationship with anxiety and aggression among this study population There is urgent need for HIV care providers to screen for suicide and link to mental health services.

## Data Availability

The raw data supporting the conclusions of this article will be made available by the authors, without undue reservation.

## Footnotes

1 Ten leading causes of death by age group [Internet]. 2020. Available from: https://webappa. cdc.gov/sasweb/ncipc/leadcause.html.

## Abbreviations

HIV/AIDS, human immunodeficiency virus/acquired immunodeficiency syndrome; ART, antiretroviral therapy; CD, cluster of differentiation; GBD, global burden of disease; WHO, world health organization; UNAIDS, the joint united nations program on HIV/AIDS; LMIC, low-and-middle-income countries; SD, standard deviation; PRR prevalence rate ratio; (aPRR), adjusted prevalence rate ratio.
